# Transplantation of Mesenchymal Stem Cells: A Potential Adjuvant Therapy for COVID-19

**DOI:** 10.3389/fbioe.2020.557652

**Published:** 2020-11-05

**Authors:** Yingqian Zhu, Shasha Geng, Qingqing Li, Hua Jiang

**Affiliations:** ^1^Department of Geriatrics, Shanghai East Hospital, Tongji University School of Medicine, Shanghai, China; ^2^Department of General Medicine, Shanghai East Hospital, Tongji University School of Medicine, Shanghai, China

**Keywords:** COVID-19, SARS-CoV-2, mesenchymal stem cells, ALI/ARDS, cytokine storm, anti-inflammation, immunomodulation, clinical trial

## Abstract

Severe acute respiratory syndrome coronavirus-2 (SARS-CoV-2) is the causative pathogen for coronavirus disease-2019 (COVID-19), which has posed an increasing serious public health threat. However, still there are no approved antiviral agents or vaccines available yet. Mesenchymal stem cells (MSCs) are emerging as a novel promising adjuvant therapy for the attenuation of COVID-19 based on its putative pathogenesis. MSCs may exert anti-inflammatory, immunomodulatory, anti-apoptotic, as well as regenerative effects through a series of mechanisms. Remarkably, MSCs may be resistant to virus infection, which is fundamental for the treatment of COVID-19. The beneficial therapeutic effects of MSCs have been preliminarily proved to be safe and efficacious for the treatment of COVID-19 in current clinical trials. This work aims to review the beneficial effects of MSCs in treating ALI/ARDS, which provides novel insight into the potential therapeutic strategies against COVID-19. However, further research is warranted regarding both safety and efficacy of MSCs.

## Introduction

In the late December 2019, a newly discovered coronavirus, severe acute respiratory syndrome coronavirus-2 (SARS-CoV-2) caused an outbreak of coronavirus disease-2019 (COVID-19), which is a strongly infectious pneumonia already rapidly evolving into a worldwide pandemic (Wang C. et al., 2020; Zhu et al., 2020). The typical clinical characteristics of infected patients are fever, cough, dyspnea, lymphocytopenia, and abnormal lung shadows on chest computed tomography (CT) (Guan et al., 2020; Huang et al., 2020; Xu X. et al., 2020). Some severe cases develop the acute respiratory distress syndrome (ARDS)/acute lung injury (ALI) and multiple organ failure, ultimately leading to the fatal deaths (Yang et al., 2020). SARS-CoV-2 can be transmitted via respiratory droplets or direct contact (Lai et al., 2020). It is highly transmissible among human with the basic reproduction number (R0) estimated to be around 2.2 and even more (Lai et al., 2020; Liu Y. et al., 2020; Riou and Althaus, 2020). The high infectivity and virulence of SARS-CoV-2 lead to the number of daily reported deaths and infected cases of COVID-19 progressively increasing. As of 30 August 2020, the outbreak of COVID-19 has rapidly spread worldwide involving more than 24 million confirmed cases and 838,924 deaths (World Health Organization, 2020). COVID-19 has posed a grave threat to global public health, unfortunately, to date, there is no effective vaccines to prevent the spread of COVID-19, and the specific antiviral agent against SARS-CoV-2 is still lacking (Liu C. et al., 2020).

Mesenchymal stem cells (MSCs) are a non-hematopoietic pluripotent adult cell population with high potentials of self-renewal and multilineage differentiation (Han et al., 2019). They can be easily purified from multiple different tissue sources, such as human umbilical cord, bone marrow, adipose tissue, dental pulp, and other sources (Naji et al., 2019). In addition to being easily accessible, MSCs can be stored and repetitively expanded for therapeutic usage within a relatively short period (Golchin et al., 2019, 2020). More importantly, MSCs only express low levels of major histocompatibility complex class I (MHC I) but they are negative for MHC II expression. The characteristics of limited immunogenicity and no major ethical concerns make MSCs ideal candidates for allogeneic cell-based therapy (Jiang and Xu, 2020). Previous research has identified the positive effects of MSCs in alleviating ALI and ARDS (Wilson et al., 2015; Geiger et al., 2017; Matthay et al., 2019). Therefore, in the context of COVID-19, we summarized the latest findings indicating that MSCs may have promising adjuvant function in treating COVID-19 induced pneumonia. We aim to provide the up-to-date understanding of therapeutical mechanism of MSCs as well as the current clinical trials of MSCs on COVID-19, hoping to suggest novel insights into the management of this highly contagious disease.

## The Pathophysiology of COVID-19

The causative agent of COVID-19, SARS-CoV-2, is a new member in the coronavirus family. It is an enveloped, positive-sense, and single-stranded RNA virus with a genome of approximately 29 kb in size, which can encode the structural and non-structural proteins (Chan et al., 2020; Wu F. et al., 2020). Like all coronaviruses, SARS-CoV-2 consists of four major structural proteins, spike (S), envelop (E), membrane (M), and nucleocapsid (N) (Yuki et al., 2020). Spike is a kind of highly glycosylated protein protruding from the virion surface, mediating angiotensin-converting enzyme 2 (ACE2) receptor recognition and host cell entry (Jin et al., 2020). The pathogenesis of SARS-CoV-2 infection starts with the recognition of zinc metallopeptidase ACE2 by spike protein to gain cell entry. ACE2 has been identified as the cellular receptor for SARS-CoV-2, which is broadly expressed in multiple organs like lung, upper respiratory track, heart, brain, kidney, ileum, and bladder (Zou et al., 2020). Particularly, the expression of ACE2 is abundant in lung alveolar epithelial cells (Zou et al., 2020), which may explain the devastating damage of lung and rapid development of acute respiratory dysfunction (Zhang and Baker, 2017). Once binding to ACE2, SAR-CoV-2 enters alveolar cells and rapidly replicates. The process of cell entry involves the cleavage and priming of spike protein induced by host transmembrane serine protease-2 (TMPRSS2), which allows the fusion between viral envelope and cellular membranes (Gkogkou et al., 2020). Thereafter the virus disassembles and viral genome is released into the cytoplasm of host cells. During SAR-CoV-2 infection, binding of spike protein to ACE2 has been proposed to resulting in ACE2 shedding from cell surface (Bosso et al., 2020). ACE2 is a member of renin–angiotensin system (RAS), acting as a key negative regulator of ACE via degrading angiotensin (Ang) II into Ang-(1-7) (Zhang and Baker, 2017). Therefore, the loss of ACE2 activity ultimately leads to ALI as a consequence of increased activity of RAS, which may explain the progressive lung inflammatory responses and enhanced pulmonary vascular permeability (Elgendy and Pepine, 2020; Lopes et al., 2020).

The symptom of SAR-CoV-2 infected patients ranges from mild cough to ALI and in some cases ARDS (Chen N. et al., 2020). Patients with severe pneumonia rapidly progressed to ARDS and multiple organ failure, which often resulted in death in a short time. Typically, histological analyses of lungs from COVID-19 patients succumbed to SAR-CoV-2 infection showed pulmonary edema, formation of hyaline membranes, bilateral diffuse alveolar damage with interstitial inflammatory infiltrates, demonstrating the feature of evident inflammatory responses and existence of ARDS (Liu Q. et al., 2020; Xu Z. et al., 2020). The excessive release of cytokines associated with significant inflammatory responses could lead to cytokine storm that considered to be the presumable causal factor for ARDS (Lai et al., 2020). Severe cases tended to have significantly higher concentrations of pro-inflammatory cytokines, including interleukin-6 (IL-6), tumor necrosis factor-α (TNF-α), IL-2 IL-7, IL-10, granulocyte colony stimulating factor (GCSF), interferon-γ inducible protein 10 (IP-10), and monocyte chemoattractant protein 1 (MCP-1) (Chen G. et al., 2020; Chen N. et al., 2020; Huang et al., 2020; Zhou et al., 2020). Notably, the high levels of inflammatory cytokine profile as well as increase in neutrophil count usually indicate poor outcome of COVID-19 (Cao, 2020; Tang et al., 2020). The number of leukocytes, the levels of C-reactive protein (CRP), procalcitonin, and several inflammatory chemokines, such as CCL2 and CXCL10, were significantly elevated in severely ill patients (Guan et al., 2020; Wang D. et al., 2020; Xiong et al., 2020). Furthermore, for severe patients with COVID-19, lymphopenia was a common feature, with reduced but overactivated peripheral CD4 T cells, CD8 T cells, B cells, and natural killer (NK) cells (Huang et al., 2020; Qin et al., 2020; Tan et al., 2020; Xu Z. et al., 2020). The overactivation of T cells exhibited high concentration of cytotoxic granules in CD8 T cells and increase of Th17, suggesting the severe immune disorder (Xu Z. et al., 2020).

It is believed that the excessive production of pro-inflammatory cytokines as well as dysregulated host immune responses, ultimately causing cytokine storm, play a pivotal role in the pathogenesis of COVID-19 (Jin et al., 2020; Tang et al., 2020). The cytokine storm usually induces the deleterious clinical manifestations of ARDS and death as a consequence of massive tissue damage, apoptosis of alveolar epithelial and endothelial cells, as well as vascular leakage (Channappanavar and Perlman, 2017). In addition to the severe pulmonary injury induced by SAR-CoV-2, some patients also displayed non-respiratory symptoms such as diarrhea, heart injury, and kidney failure, which may be associated with broad expression of ACE2 in multiple human organs (Huang et al., 2020; Yang et al., 2020).

## Therapeutic Mechanism of MSCs Therapy in Treating COVID-19

**FIGURE 1:**
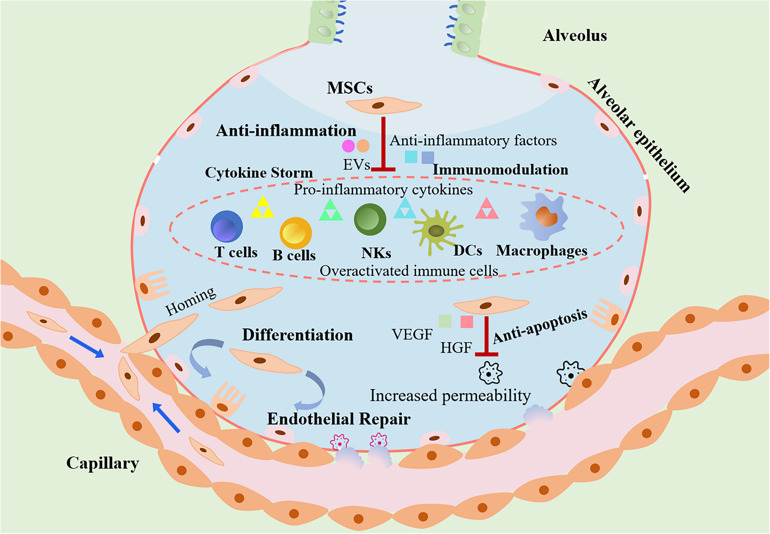
The role of MSCs transplantation as a promising therapeutic option in COVID-19 treatment. MSCs attenuate the cytokines storm through secretion of anti-inflammatory factors and regulation of the immune system. Due to loss of endothelial barrier integrity in patients with COVID-19, vascular permeability increased. MSCs can engraft to the injured sites and differentiate into lung epithelial and endothelial cells for reparation. In addition, MSCs protect host epithelial cells from apoptosis. As a consequence, the transplantation of MSCs could exert beneficial effects against ALI/ARDS induced by SARS-CoV-2 infection.

A growing evidence has shown that MSCs exert anti-inflammatory effects, inhibit excessive immune system response. More importantly, MSCs can differentiate and have been found to promote regeneration of tissue damage mediated by virus infection. MSCs may attenuate the SAR-CoV-2 associated lung injury, holding potential therapeutic effects on the treatment of COVID-19. Figure 1 describes potential mechanisms of MSCs transplantation for the treatment of COVID-19 pneumonia.

## Anti-Inflammation and Immunomodulation

The exuberant inflammatory responses induced by SAR-CoV-2 that are always accompanied by cytokine storm lead to progressive ALI/ARDS and even death (Huang et al., 2020). Inflammatory modulation has been considered to be the key in the successful control of COVID-19. Although corticosteroid exerts potent anti-inflammatory effect, the use of corticosteroid therapy was relatively restricted with regard to the delay in virus clearance (Ling et al., 2020). The advancing therapeutic interventions with anti-inflammatory effects are urgently needed. MSCs are capable of reducing inflammation and ameliorating cytokine storm, thereby protecting lung epithelial cells from death during ALI/ARDS and decreasing mortality of COVID-19 (Ji et al., 2020). Preclinical studies have showed that MSCs could ameliorate acute alveolar injury and inflammation in the mouse model of ALI/ARDS (Zhang et al., 2017, 2018; Horie et al., 2018). MSCs can be systemically infused and then migrate to the injured sites. Once lodging in the lungs, MSCs could release a wide variety of secretome or extracellular vesicles (EVs) to exert the anti-inflammatory effects. It has been reported that after treatment of MSCs in patients of COVID-19, the peripheral lymphocytes increased while CRP levels decreased (Leng et al., 2020). Remarkably, the COVID-19 patients who received MSC-based therapy displayed a reduction in the level of TNF-α, a pro-inflammatory cytokine, and an increase in the levels of an anti-inflammatory mediator IL-10 (Leng et al., 2020).

The anti-inflammatory effects exerted by MSCs may be partly through paracrine pathways, which is manifested by the presence of several soluble anti-inflammatory factors secreted by MSCs. MSCs can release of anti-inflammatory cytokines and trophic factors such as transforming growth factor-β (TGFβ), vascular endothelial growth factor (HGF), vascular endothelial growth factor (VEGF), epidermal growth factor (EGF), and brain-derived neurotrophic factor (BDNF). The highly expressed factors in MSCs suggested that the immunomodulation of MSCs can be actively maintained by continuing cytokine production (Leng et al., 2020). Notably, young MSC-derived EVs that contain specific mRNA and peptides with anti-inflammatory properties can migrate to injured sites to alleviate ALI/ARDS (Monsel et al., 2015). As a consequence, MSCs may recover the inflammatory microenvironment of lungs and alleviate the SARS-CoV-2-derived ALI/ARDS, and even have a potential effect on the treatment of subsequent pulmonary fibrosis.

The invasion of SAR-CoV-2 in the host initiates the innate immune responses and subsequent adaptive immune responses. T cells responses are driven by antigen-presenting cells (APCs) via presenting viral antigens to T cells. APCs present the virus-derived components to T cells and trigger immune responses to clear the viruses. The antiviral immunity is important for the viral clearance, but they also cause significant damage to the alveolar epithelium and lung capillary endothelium (Herold et al., 2015). The immune system is over-activated when killing the viruses, but it produces high levels of inflammatory cytokines, ultimately worsening lung injury and inducing organ damage. The activation of immune system is demonstrated by the lymphopenia and overactivation of T cells in the peripheral blood of COVID-19 patients (Huang et al., 2020; Xu Z. et al., 2020). Therefore, immunomodulatory therapy is beneficial for SAR-CoV-2 infected patients. MSCs possess immunomodulatory property via inhibiting the innate immune responses as well as adaptive immune responses (Hosseinikia et al., 2017). MSCs are capable of regulating the activity of T cells, B cells, macrophages, monocytes, dendritic cells (DCs), and NK cells through cell–cell contact and their secretome (Haddad and Saldanha-Araujo, 2014; Li and Hua, 2017). T cells and B cells are the hallmark effector cells in the adaptive immune system. Several lines of evidence showed that MSCs could inhibit the proliferation and activation of T cells simulated by inflammatory cytokines (Ren et al., 2008; Li and Hua, 2017; Kadle et al., 2018; Hu and Li, 2019). MSCs have been found to exert immunosuppressive functions via recruiting and promoting the generation of regulatory T cells (Tregs) from CD4 and CD8 T cells (Rashedi et al., 2017). Similarly, MSCs are capable of inducing cell cycle arrest in B cells with reduced antibody-producing ability and impaired plasma cell generation (Li and Hua, 2017). Furthermore, MSCs can inhibit the differentiation of B cells by the induction of regulatory B cells (Bregs) (Franquesa et al., 2015). As to the effects of MSCs on innate immunity, MSCs inhibit the responses of a group of major cells that involved in the innate immunity. MSCs have been shown to suppress DCs maturation (Spaggiari et al., 2009; Anderson et al., 2017), inhibit NK cells cytotoxic activity (Li et al., 2015), as well as stimulate conversion of macrophage into regulatory macrophages with anti-inflammatory properties (Uccelli and de Rosbo, 2015; Sava et al., 2020). Aside from exerting immunoregulatory effects, MSCs are capable of escaping the immune surveillance. The peculiar immune modulation and immune privilege render MSCs the promising agents for the management of COVID-19.

## Regeneration and Differentiation

The lung vascular endothelium is the first barrier to prevent inflammatory cells and proteins from the vasculature infiltrating into the alveoli and interstitial space of the lung (Bhattacharya and Matthay, 2013). Loss of endothelial barrier integrity results in the increased vascular permeability, leading to influx of protein-rich edema fluid into air spaces. These pathological changes have been demonstrated by the histological examination of COVID-19 patients, which presented diffuse alveolar damage and hyaline membrane formation with pulmonary edema, the evident signs of ARDS (Xu Z. et al., 2020). It is essential to restore the injured endothelial barrier properties for the treatment of SAR-CoV-2 induced ALI/ARDS. A growing number of evidences have suggested that MSCs replenish the pulmonary permeability the via paracrine mechanisms. MSCs are capable of secreting VEGF, which is a trophic factor responsible for the growth and survival of endothelial cells in the lung, thereby the administration of MSCs could exert beneficial effects against ALI/ARDS by protecting impaired alveolar (Chang et al., 2014). Similarly, MSCs can secrete HGF which plays a critical role in maintaining endothelial barrier properties (Hu et al., 2016).

Moreover, MSCs can migrate and engraft in the injured lung sites and have a potential effect to differentiate into lung epithelial and endothelial cells, which lead to deceased destructive changes, attenuated diffuse edema as well as improved respiratory function (Maron-Gutierrez et al., 2014; Han and Mallampalli, 2015). Notably, surfactant associated protein A (SPA) and surfactant associated protein C (SPC) are highly expressed in MSCs, indicating that MSCs might differentiate to alveolar type II cells (Leng et al., 2020). In this regard, MSCs directly restore the pulmonary endothelial barrier during lung injury, holding promise as a novel adjuvant therapy for the treatment of COVID-19.

## Anti-Apoptosis

Apoptosis plays a crucial role in maintenance the homeostasis between cell death and survival (Chambers et al., 2018). However, the excessive and inadequate apoptosis can lead to the pathogenesis of diseases. It has been suggested that ALI/ARDS is associated with a dysbalanced lung cell apoptosis (Kosutova et al., 2018; Wu Y. et al., 2020). The pulmonary epithelial cells are exposed and susceptible to multiple environmental stress and internal responses, in particular, virus infection is an important stimulus that triggers apoptosis of epithelial cells (Yeung et al., 2016; Khatri et al., 2018). MSCs have potential effects in regulating the apoptosis of host epithelial cells via paracrine mechanisms. MSCs could release EVs; the MSCs-derived EVs significantly inhibit the apoptosis of lung epithelial cells induced by virus infection (Khatri et al., 2018). Furthermore, it has been found that MSCs-derived conditioned medium has a protective effect by promoting proliferation and inhibiting apoptosis of lung epithelial cells (Peng et al., 2019). Future research will hopefully help elucidate the exact mechanism.

## Concerns on MSCs-Based Therapy

Previous findings have documented that MSCs are susceptible to a variety of viruses, including influenza viruses (Khatri and Saif, 2013; Khatri and Chattha, 2015), bursal disease virus (Khatri and Sharma, 2009), hepatitis B virus (Ma et al., 2011), and human herpesvirus (Bortolotti et al., 2018), among others. The virus-infected MSCs could lead to the progressive pathology induced by viruses via directly or indirectly affecting other immune cells (Khatri and Saif, 2013). Thereby, concerns have been raised regarding that the therapeutic MSCs infused to COVID-19 patients could be infected by SARS-CoV-2.

It has been well acknowledged that ACE2 is the major host cell receptor for SARS-CoV-2 entry. ACE2 receptors as well as TMPRSS2 are highly distributed on host cells, particularly the lung alveolar type II cells and endothelial cells, are critical for the progression of COVID-19. To address this concern, RNA-seq has revealed that MSCs were ACE2 and TMPRSS2 negative, which could be free from COVID-19 infection (Leng et al., 2020). Encouragingly, some studies have indicated that MSCs can generally resist viral infection via expressing interferon stimulated genes (ISGs) (Wu et al., 2018). MSCs could intrinsically express various ISGs that are known for the antiviral defense. Particularly, the protein members of interferon induced transmembrane family (IFITM) can forestall viral infection before the virus traversing the lipid bilayer of cell. With the antiviral activities of ISGs, some pathogenic viruses like SARS coronavirus can be effectively defensed (Bailey et al., 2014). The antiviral mechanism of MSCs could be possibly attributed to the following two activities: MSCs can elevate the levels of ISGs, which are MSC-specific and acting as mediators of antiviral protection; involving the secondary response to interferon, which can lead to ISG induction and broad viral resistance (Khoury et al., 2020). The properties of virus resistance provide new insight into the therapeutic potential of MSCs in the treatment of COVID-19.

## Clinical Application of MSCs for COVID-19

On the basis of their extensive capability, MSCs have been utilized for the first time for the intervention of COVID-19 on 23 January 2020. In this clinical study reported by Dr. Zhao et al. (Leng et al., 2020), a total of seven patients with confirmed COVID-19 received single intravenous dose of clinical grade MSCs, with one displaying critically severe type, four severe types, and two common type, while three severe types in controls received placebo. The number of cells infused was 1 × 10^6^ cells per kilogram of weight. Safety and efficacy of intravenous MSCs transplantation were evaluated. There were no adverse events reported including acute infusion-related or allergic reactions post infusion. Moreover, no secondary infections delayed reaction induced by MSCs were observed. With regard to the efficacy of MSC-based therapy, the majority of patients were negative for SARS-CoV-2 nucleic acid test over a short period post MSCs administration. The possible mechanism underlying may be attributable to beneficial advantage of MSCs. MSCs inhibit the overactivation of immune system through increasing regulatory DCs and decreasing cytotoxic T cells. It is notable that MSCs prevent the cytokine storm via secreting anti-inflammatory factors. In addition, MSCs result in overall functional improvement and significant recovery in pneumonia infiltration. Recently, a parallel assigned, controlled, and phase I clinical trial was conducted to evaluate the safety and efficacy of human umbilical cord-derived MSCs.

Aside from standard COVID-19 treatment regimens, nine of 18 patients with moderate and severe COVID-19 intravenously received three doses of MSCs (3 × 10^7^ cells per infusion). Due to the small sample size, there was no comparative analysis between the two groups, but the patients in the MSCs group displayed a reduction of serum IL-6. Moreover, this trial demonstrated that transplantation of MSCs in patients with COVID-19 was safe and no serious adverse events were reported (Meng et al., 2020). In addition, a case report has described the clinical application of human umbilical cord-derived MSCs in a patient with severe COVID-19 pneumonia, who was intravenous injected with 1 × 10^6^ cells/kg. After MSCs transplantation, the symptoms like high fever and shortness of breath disappeared. Additionally, the pulmonary function as well as chest CT imaging was greatly ameliorated. This report provided the available evidence on the safety and efficacy of MSCs as adjunctive therapy for the treatment of COVID-19 (Zhang et al., 2020). The clinical study published and case report have demonstrated that intravenous transplantation of MSCs was safe and effective, whereas more clinical trials with larger samples are warranted for more convincing evidence.

To fully understand the current clinical research utilizing MSCs, a search for registered trials in *ClinicalTrials.gov* was performed, which identified a total of 51 ongoing or completed interventional trials to date. The most common sources of MSCs are human umbilical cord-derived MSCs, successively followed by bone marrow-derived MSCs, adipose-derived MSCs, dental pulp-derived MSCs, placenta-derived MSCs, and olfactory mucosa-derived MSCs. Notably, the overwhelming majority of clinical trials transplant MSCs by intravenous injection, except for one trial delivering MSCs through intramuscular injection, and the remaining two trials using MSCs-derived exosomes via aerosol inhalation. Despite well designed, the majority of current clinical trials are in phase 1 or 2, an initial stage with small samples. To date, there is only one completed trial and approximately half are recruiting participants, while the rest of trials are not yet recruiting. According to the data that have been published, it is likely to believe that transplantation of MSCs for the treatment of COVID-19 is safe and effective. An overall summary of trial characteristics is shown in Table 1 and details of each included trials are provided in Supplementary Table 1.

**TABLE 1:** Characteristics of clinical trials.

	**Number**	**Percent (%)**
**Mesenchymal stem cell type**		
Umbilical cord-derived MSCs	22	43.14
Bone marrow-derived MSCs	11	21.57
Adipose-derived MSCs	6	11.76
Dental pulp-derived MSCs	2	3.92
Placenta-derived MSCs	1	1.96
Olfactory mucosa-derived MSCs	1	1.96
NR	8	15.69
**Administration route**		
Intravenous injection	48	94.12
Intramuscular injection	1	1.96
Aerosol inhalation	2	3.92
**Trial phase**		
Phase 1	15	29.41
Phase 2	15	29.41
Phase 3	2	3.92
Phase 1/2	15	29.41
Phase 2/3	1	1.96
NA	3	5.89
**Sample size**		
1–20	15	29.41
21–50	20	39.22
51–100	11	21.57
101–500	4	7.84
501–1000	1	1.96
**Number of arms**		
1	9	17.65
2	33	64.71
≥3	9	17.65
**Treatment allocation**		
Randomized	37	72.55
Non-randomized	6	11.76
NA	8	15.69
**Masking (blinding)**		
Open label	23	45.10
Single blind	2	3.92
Double blind	6	11.76
Triple blind	10	10.61
Quadruple blind	10	10.61
**Endpoint classification**		
Safety/efficacy study	36	70.59
Efficacy study	14	27.45
Safety study	1	1.96
**Trial overall status**		
Recruiting	27	52.94
Not yet recruiting	18	35.29
Active, not recruiting	5	9.81
Completed	1	1.96

*NR, not reported; NA, not applicable.*

## Therapeutic Effects of MSCs in Preclinical Models of Respiratory Virus Induced Lung Injury

Although the evidence from the clinical practice settings is relatively limited, there are a growing number of pre-clinical studies suggesting the potential efficacy of MSC-based therapy in treating respiratory viral lung infections. A pre-clinical study has investigated therapeutic effects of MSCs on ALI induced by avian influenza virus (H5N1) infection *in vitro* and *in vivo*. Encouragingly, the enhanced alveolar epithelium’s protein permeability and reduced alveolar fluid transport of human alveolar epithelial cells, caused by H5N1 influenza virus, were significantly prevented by coculture with MSCs in vitro. A total of 5 × 10^5^ MSCs in 100 μL PBS were intravenously infused into the H5N1 virus-infected mice at day 5 p.i., aged mice treated with MSCs had significantly greater survival and body weight, with ameliorated lung edema and decreased pro-inflammatory and chemokines, which prevented H5N1-associated ALI *in vivo* (Chan et al., 2016). Similarly, a recent study also documented the therapeutic efficacy of human umbilical cord-derived MSCs in a mouse model of influenza H5H1 associated ALI. Moreover, it revealed that MSCs derived from human umbilical cord outperformed MSCs from bone marrow, partially due to greater secretion of angiopoietin 1 and HGF (Loy et al., 2019). In contrast, a previous study reported that MSCs administrated intravenously twice failed to attenuate influenza H1N1 associated ALI in mice. However, MSCs can be protective against thrombocytosis mediated by H1N1 and reduce the lung viral load modestly (Gotts et al., 2014). Therefore, despite the potent therapeutic effects in several models of inflammatory diseases, further pre-clinical studies are highly needed to investigate the effects of MSCs transplantation in lung injury models induced by SAR-CoV-2 infection.

## Conclusion

Remarkably, the study provides novel insights regarding potential roles of MSCs in SARS-CoV-2 associated lung injury. On the basis of the reported beneficial effects of MSCs, such as anti-inflammatory, immunomodulatory, regenerative, and anti-apoptotic properties, MSC-based therapy may contribute to reduce the severity and lethality of COVID-19. The current evidence from clinical observation, albeit still insufficient, has preliminarily indicated that administration of MSC is evolving to be a potential adjuvant therapy for COVID-19. However, further studies, including clinical trials and experimental researches, are urgently needed to clarify the underlying mechanisms and assist the decision making in the midst of COVID-19.

## Author Contributions

HJ conceived and supervised the manuscript. YZ, SG, and QL prepared and wrote the manuscript. All authors contributed to the manuscript revision.

## Conflict of Interest

The authors declare that the research was conducted in the absence of any commercial or financial relationships that could be construed as a potential conflict of interest.
